# Cold Water in Puerperal Convulsions

**Published:** 1852-05

**Authors:** G. W. Booth

**Affiliations:** Carrollville, Mississippi


From the Southern Medical and Surgical Journal.

*Cold Water in Puerperal Convulsions.* By G. W. Booth, M. D., of Carrollville, Mississippi.

In the year 1848, I was called early one morning to see Mrs. E. W., wife of P. W. On my arrival, I was informed that she had been in labor for several hours. She was about 18 years of age, short stature, and of ruddy appearance. This was her first pregnancy, and I was informed by the old midwife who was in attendance that the labor appeared to progress favorably till a short time before I was called in. The patient's friends had discovered some convulsive twitchings about her face which alarmed them, and I was consequently summoned.

The waters had been discharged sometime before I saw her.—On examination, I found the head at the inferior strait presenting naturally, and the soft parts in a favorable condition. The pains appeared to be effective. Soon after I made an examination, the muscular twitchings about the face deepened into the most terrific general convulsions I have ever witnessed. She was delivered in a few minutes after the accession of the convulsions, of a fine healthy girl, of average size. I was in hopes, after this event, that some mitigation, at least, would take place in the symptoms. In this, however, I was disappointed, as the convulsions continued to recur as before. She remained perfectly unconscious, from the first severe paroxysm, throughout the course of the disease. I

put into vigorous play all the usual remedies for this formidable disease, but without influencing it materially. I continued this treatment till late in the evening. I now despaired of the patient's life, and announced the fact to her husband and friends. As a dernier resort, I determined to try the effect of cold water, applied by pouring it freely over the whole system. To carry this treatment into effect, I had her taken from the bed and laid on the floor, with a quilt under her and a sheet thrown over her body. I then, from a large pitcher, poured water, fresh from a well, over her, from head to feet, for several minutes. After the application of the water, I had dry clothes put on the patient, and she was replaced in bed. In the course of half an hour after this she awoke from the stupor, which had existed since morning, perfectly rational, and had no return of convulsions after the water had been used. She now had no recollection of anything that had taken place since her first convulsion, and appeared to be surprised to learn that her child was born.

There was inability to pass off her urine, and I drew off a large quantity with a catheter—the state of her bladder having been unattended to in the earlier part of her labor. I used the instrument but once, as she was able afterwards to evacuate the bladder without its aid. She had a fine "getting-up," and was as well in a few days as is common after the most favorable labors.

Mrs. W. has borne two children since, without any untoward circumstance attending either labors.

